# IMPACT OF SELF-PERCEPTION OF AGING ON THE RELATIONSHIP BETWEEN HEALTHY BEHAVIOR, STRESS, AND HEALTH IN MIDLIFE

**DOI:** 10.1093/geroni/igz038.1719

**Published:** 2019-11-08

**Authors:** Hyun-E Yeom, Eunyoung Park, Misook Jung

**Affiliations:** Chungnam National University, Daejeon, Korea, Republic of

## Abstract

Self-perception of aging may affect the interpretation of health-related changes that influence behaviors. Understanding how self-perception of aging is associated with healthy behavior, stress, and health is essential to prepare healthy aging. The purposes of this study are to examine the influence of healthy behavior on stress and subjective health status and to investigate whether self-perception of aging affects the association in midlife Koreans. This is a cross-sectional study. Data were collected through a self-administered survey using a convenience sampling of 466 midlife Koreans (mean age = 50.7, 52.4% male). The PROCESS macro for SPSS was applied for data analysis. Self-perception of aging was significantly related to stress (r= .20, p<.00), and subjective health was related to healthy behavior (r= .22, p<.00) and stress (r= -.38, p<.00). Healthy behavior predicted stress, which, in turn, affected subjective health status (index=.05, 95% CI [.01, .10]). A significant interaction between healthy behavior and self-perception of aging was found (β= -.22, p=.002), indicating that the influence of healthy behavior on stress was different depending on the individual’s self-perception of aging. It means that the impact of healthy behavior on stress was stronger in individuals with a more negative self-perception of aging. The findings demonstrate the critical role of self-perception of aging, which affects the benefits of healthy behavior on stress and subjective health status. This study highlights the importance of assessing self-perception of aging and developing cognitive behavioral interventions that contribute to modifying stereotyped beliefs about aging for better quality of life in midlife.

